# Compositional and Quantitative Insights Into Bacterial and Archaeal Communities of South Pacific Deep-Sea Sponges (Demospongiae and Hexactinellida)

**DOI:** 10.3389/fmicb.2020.00716

**Published:** 2020-04-24

**Authors:** Georg Steinert, Kathrin Busch, Kristina Bayer, Sahar Kodami, Pedro Martinez Arbizu, Michelle Kelly, Sadie Mills, Dirk Erpenbeck, Martin Dohrmann, Gert Wörheide, Ute Hentschel, Peter J. Schupp

**Affiliations:** ^1^RD3 Marine Symbioses, GEOMAR Helmholtz Centre for Ocean Research Kiel, Kiel, Germany; ^2^Institute for Chemistry and Biology of the Marine Environment (ICBM), University of Oldenburg, Oldenburg, Germany; ^3^German Center for Marine Biodiversity Research, Senckenberg Research Institute, Wilhelmshaven, Germany; ^4^National Institute of Water and Atmospheric Research, Ltd., Auckland, New Zealand; ^5^National Institute of Water and Atmospheric Research, Ltd., Wellington, New Zealand; ^6^Department of Earth and Environmental Sciences, Paleontology & Geobiology, Ludwig-Maximilians-Universität München, Munich, Germany; ^7^GeoBio-Center, Ludwig-Maximilians-Universität München, Munich, Germany; ^8^Bayerische Staatssammlung für Paläontologie und Geologie, Munich, Germany; ^9^Christian-Albrecht University of Kiel, Kiel, Germany; ^10^Helmholtz Institute for Functional Marine Biodiversity at the University of Oldenburg (HIFMB), Oldenburg, Germany

**Keywords:** 16S rRNA amplicons, archaea, bacteria, Demospongiae, Hexactinellida, Porifera, quantitative real-time PCR (qPCR), South Pacific Ocean

## Abstract

In the present study, we profiled bacterial and archaeal communities from 13 phylogenetically diverse deep-sea sponge species (Demospongiae and Hexactinellida) from the South Pacific by 16S rRNA-gene amplicon sequencing. Additionally, the associated bacteria and archaea were quantified by real-time qPCR. Our results show that bacterial communities from the deep-sea sponges are mostly host-species specific similar to what has been observed for shallow-water demosponges. The archaeal deep-sea sponge community structures are different from the bacterial community structures in that they are almost completely dominated by a single family, which are the ammonia-oxidizing genera within the Nitrosopumilaceae. Remarkably, the archaeal communities are mostly specific to individual sponges (rather than sponge-species), and this observation applies to both hexactinellids and demosponges. Finally, archaeal 16s gene numbers, as detected by quantitative real-time PCR, were up to three orders of magnitude higher than in shallow-water sponges, highlighting the importance of the archaea for deep-sea sponges in general.

## Introduction

Marine sponges (Porifera) host a broad range of microorganisms including bacteria, archaea, eukaryotes, and viruses and are therefore considered holobionts (Webster and Thomas, 2016; Pita et al., 2018). Sponges are integral parts of the marine ecosystem as they couple pelagic and benthic ecosystems by virtue of their massive filter-feeding capacities (Vogel, 1977; de Goeij et al., 2017; Pita et al., 2018). Sponge-associated symbionts perform critical functions for their host, including among others, the provision of nutrients (particularly, for nitrogen and carbon) and chemical defense which affect host health and functioning (Slaby et al., 2019). The microbial consortia of sponges are represented by diverse prokaryotic communities with ≥ 63 phyla having been found in sponges so far (Thomas et al., 2016; Moitinho-Silva et al., 2017b). These prokaryotic communities show sponge species-specific patterns that differ in richness, diversity, and structure from the prokaryotic seawater communities. The composition of the sponge symbiont consortia is shaped by host taxonomy in that sponge species have species-specific prokaryotic communities (Pita et al., 2013; Easson and Thacker, 2014; Thomas et al., 2016; Steinert et al., 2017; Cárdenas et al., 2019). Machine learning provided evidence that the dichotomy between high microbial (HMA) and low microbial abundance (LMA) sponges is a main driver of the sponge-associated community patterns (Moitinho-Silva et al., 2017c). Several prokaryotic phyla and taxa were identified as indicator taxa for either one of the two abundance states, such as Chloroflexi (e.g., SAR202, Caldilineaceae), Acidobacteria (e.g., Solibacteres, PAUC37f, Sva725), Poribacteria or Actinobacteria (e.g., Acidimicrobiia) for HMA sponges, or alternatively, Proteobacteria (e.g., Gammaproteobacteria), Bacteroidetes (e.g., Flavobacteriia), and Planctomycetes (e.g., Planctomycetia) for LMA sponges. The global sponge microbiome data revealed further community features, such as the dominance of specialists and generalists within the symbiont communities, a stable core microbiota, and community structure and functional modularity, with abiotic factors influencing the overall sponge microbiota and biotic factors the prokaryotic core (Thomas et al., 2016; Lurgi et al., 2019).

Most sponge microbiome studies have focused on demosponges that were collected from shallow coastal sites in temperate, subtropical, and tropical sampling locations (e.g., Schmitt et al., 2012; Moitinho-Silva et al., 2014; Naim et al., 2014; Thomas et al., 2016; Steinert et al., 2017; Helber et al., 2019). Considering that the deep-sea is the largest, still relatively underexplored habitat on earth, comparably few studies have been conducted on sponges from remote deep-sea or cold-water locations (e.g., Jackson et al., 2013; Kennedy et al., 2014; Reveillaud et al., 2014; Borchert et al., 2017). Antarctic shallow cold-water demosponges host dominant bacterial taxa that are known to be sponge-associated (Webster et al., 2004; Rodríguez-Marconi et al., 2015; Cárdenas et al., 2018, 2019), and Antarctic deep-water demosponges display high levels of host-specificity (Steinert et al., 2019). In addition, sponges from classes other than Demospongiae, i.e., Hexactinellida, Calcarea, and Homoscleromorpha, are still only poorly covered by 16S rRNA gene sequencing approaches (but see Xin et al., 2011; Tian et al., 2016) and are consequently still underrepresented in global sponge microbiome surveys. Finally, most microbiome studies have used bacterial universal 16S rRNA gene primers, hence the sponge-associated archaeal communities have largely been missed (but see, e.g., Webster et al., 2010; Schmitt et al., 2012; Thomas et al., 2016; Moitinho-Silva et al., 2017a; for exceptions, covering both bacterial and archaeal diversity). In North Atlantic deep-sea sponges, Archaea were previously proposed to be important members of a potential deep-sea specific sponge microbial community (Jackson et al., 2013; Kennedy et al., 2014). Archaeal predominance has also been observed in one Arctic deep-water demosponge species (Pape et al., 2006). Ammonia oxidizing archaea (AOA) were identified as the main contributors of nitrification within the cold-water sponge hosts (Hoffmann et al., 2009; Radax et al., 2012; Li et al., 2014). One recent study underlined the importance of AOA in deep-sea sponges using metagenomic data obtained from one glass sponge (Tian et al., 2016).

Our present study aims to contribute to resolving sponge-prokaryote relationships from understudied habitats by exploring both bacterial and archaeal communities in demosponges and hexactinellids, which were collected from meso-, bathypelagic, and abyssal depths in the South Pacific Ocean offshore New Zealand. We investigate whether current sponge-microbiota paradigms hold up for those remotely collected and partially novel sponge species. We address the following questions: (a) are general principles of the Demospongiae microbiota also present in Hexactinellida prokaryotic communities, (b) do these sponge-bacterial community principles also apply to the archaeal community structures in marine sponges, and (c) can we compare the observed deep-sea sponge microbiota to the tropical/warm water sponge microbiota? In addition to these main questions, which we addressed using two high-throughput 16S rRNA gene sequencing data libraries, we also applied quantitative PCR (qPCR) to a subset of the sponge specimens, because quantitative data are frequently missing in sponge microbiome studies.

## Materials and Methods

### Sample Processing and Sponge Taxonomy

**FIGURE 1:**
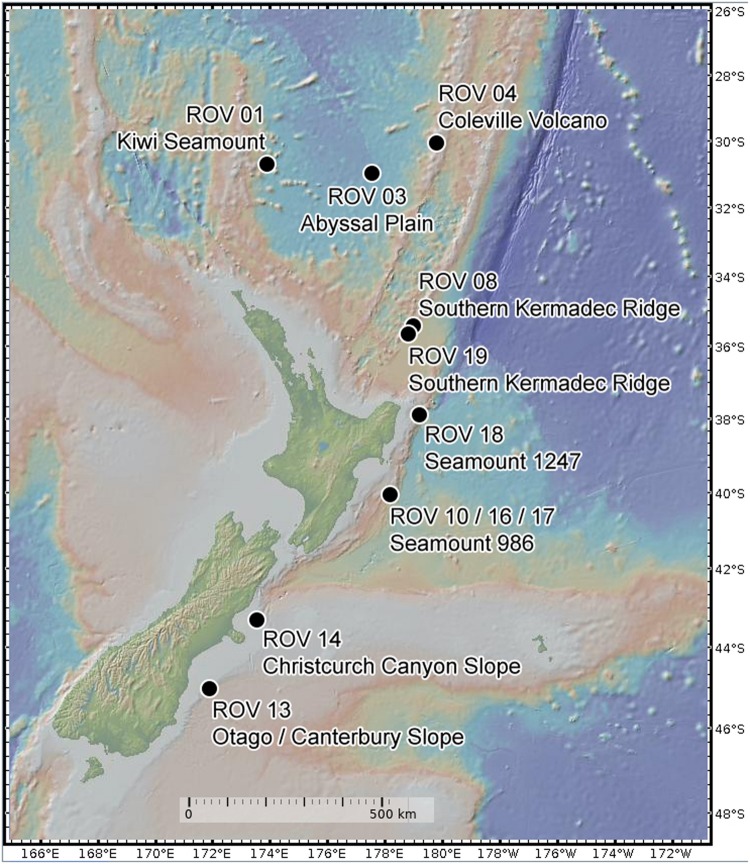
Sampling site map from New Zealand. Dots indicate the collection site with ROV dive number and name of the underwater site.

During the SO254 expedition of the research vessel “*Sonne”* in the South Pacific Ocean south- to northeast around New Zealand in February 2017, over 200 sponge specimens, of 96 sponge taxa comprising the Porifera classes Demospongiae and Hexactinellida, were collected by using a remotely operated vehicle (ROV “*Kiel 6000*”^1^). Of these 200 sponges, only species collected in at least triplicate were considered in the microbiome analysis. This criterion reduced the investigated sponge diversity to 45 specimens and 13 species. Depths of the nine collecting sites of the sponges included in the analysis ranged from 472 to 4160 m (Figure 1, Table 1 and Supplementary Table S1). Sponges were placed in coolers with ice packs upon removal from the bioboxes of the ROV. Briefly, the sponges were photographed and subsequently dissected (Supplementary Figure S1). Small tissue sub-samples were instantly frozen for molecular analyses, while larger tissue samples were stored in ethanol (80%) as vouchers for morphological identification. All samples were stored at −80°C until further processing (see section “Supplementary Material”). In addition, nine triplicate seawater samples were collected at several sponge sampling sites from the same depths using Niskin bottles attached to the ROV. Approximately 2000 ml of each seawater sample was filtered using PVDF filter membranes (0.22 μm pore size and ø 47 mm) and stored at −80°C until further processing. Sponge species were identified by morphological and molecular taxonomic methods (see section “Supplementary Material and Table 1”). Sponge tissue vouchers were stored in three collections, NIWA Invertebrate Collection in New Zealand, ICBM Environmental Chemistry Collection, University of Oldenburg, Germany and Ludwig-Maximilians-Universität, in Germany and are available on request (Supplementary Table S1).

**TABLE 1:** Sponge sample list, including taxonomy from class to species, number of replicates, sampling sites, and sampling depths.

**Class**	**Order**	**Family**	**Genus and species**	**Replicates**	**Sampling locations**	**Sampling depths**
Demospongiae	Axinellida	Stelligeridae	*Paratimea* sp. indet.	4	Coleville Volcano	472–532
Demospongiae	Haplosclerida	Halichondriidae	*Halichondria* sp. indet.	3	Seamount 986	892–899
Demospongiae	Poecilosclerida	Latrunculiidae	*Latrunculia morrisoni*	3	Otago/Canterbury Slope, Christchurch Canyon Slope	595, 670–706
Demospongiae	Tetractinellida	Geodiidae	*Geodia vaubani*	4	Southern Kermadec Ridge, Seamount 986	1172–1216, 802
Demospongiae	Tetractinellida	Geodiidae	*Penares turmericolor*	4	Southern Kermadec Ridge	1187–1191
Demospongiae	Tetractinellida	Pleromidae	*Pleroma turbinatum*	3	Coleville Volcano	472–497
Hexactinellida	Lyssacinosida	Euplectellidae	*Bolosoma cyanae*	4	Southern Kermadec Ridge	1149–1167
Hexactinellida	Lyssacinosida	Euplectellidae	*Corbitella plagiariorum*	4	Seamount 986	770–802
Hexactinellida	Lyssacinosida	Euplectellidae	*Regadrella okinoseana*	4	Seamount 986	774–896
Hexactinellida	Lyssacinosida	Euplectellidae	*Saccocalyx tetractinus*	3	Seamount 1247, Abyssal Plain	1352–1457, 4160
Hexactinellida	Lyssacinosida	Leucopsacidae	*Leucopsacus distantus*	3	Seamount 986	792–896
Hexactinellida	Lyssacinosida	Rossellidae	Lanuginellinae gen. et sp. indet.	3	Seamount 986	802–893
Hexactinellida	Sceptrulophora	Aphrocallistidae	*Aphrocallistes beatrix*	3	Kiwi Seamount, Seamount 986	759–793

*For a detailed list see Supplementary Table S1.*

### Bacterial and Archaeal 16S rRNA Gene Amplicon Sequencing and Processing

Initially DNA of sponges (three to four replicates per taxon) and seawater samples was extracted using the DNeasy Power Soil Kit (Qiagen) on approximately 0.25 g of sponge tissue or half a seawater filter. After the quality and quantity of the extracts had been checked (by Nanodrop and gel electrophoresis after a PCR with universal 16S rRNA gene primers), a one-step PCR was performed for amplification of the bacterial V3 to V4 variable regions (primer pair 341F 5′-CCTACGGGAGGCAGCAG-3′ and 806R 5′-GGACTACHVGGGTWTCTAAT-3′) and archaeal V4 to V6 variable regions (primer pair Uni519F 5′-CAGCMGCCGCGGTAA-3′ and 1000R 5′-GGCCATGCACYWCYTCTC-3′) of the 16S rRNA gene (Øvreås et al., 1997; Gantner et al., 2011; Takahashi et al., 2014). A quality check by gel electrophoresis, normalization, and pooling was performed on the amplicon libraries before independent sequencing of the bacterial and archaeal libraries on a MiSeq platform (MiSeqFGx, Illumina) using the v3 chemistry. This resulted in one archaeal and one bacterial 16S rRNA gene amplicon libraries (for detailed amplicon libraries preparation methods see section “Supplementary Material”). The raw data have been deposited in the Sequence Read Archive with BioProject number: PRJNA552490 (bacterial libraries) and PRJNA552540 (archaeal libraries).

Amplicon sequences were processed using QIIME2-2018.11 (Boylen et al., 2018). Bacterial and archaeal libraries were processed and analyzed in parallel applying the same plugin commands if not stated otherwise. Due to quality reasons only the bacterial and archaeal forward amplicon libraries (i.e., single-end) were used in this study. After the import of demultiplexed single-end fastq files via *qiime import*, primers were trimmed using *qiime cutadapt trim-single*. The QIIME2 plugin DADA2 (*qiime dada2 denoise-single*) was used for the detection and correction of Illumina-generated amplicon sequence data and to generate amplicon sequence variants (ASVs) using the following parameters for both libraries: *–p-trim-left 0* and *–p-trunc-len 250*. Resulting bacterial and archaeal 16S rRNA gene representative ASV sequences were used to calculate phylogenetic ASV trees for subsequent analyses using the *qiime phylogeny align-to-tree-mafft-fasttree* plugin. Bacterial and archaeal primer-specific trained Naive Bayes taxonomic classifiers^2^, using the SILVA 132 release files^3^, were used to classify the representative ASV sequences (*qiime feature-classifier classify-sklearn*). Before subsequent analyses, the complete amplicon datasets, including all available Demospongiae, Hexactinellida, and seawater sample groups, where divided into additional sample group-specific datasets via the *qiime feature-table filter-samples* plugin.

The generated exact sequence variants, or amplicon sequence variants (i.e., ASVs), are used as substitute for the commonly used operational taxonomic units (OTUs) clusters of sequencing reads by applying the QIIME2 implemented DADA2 algorithm (Callahan et al., 2016; Nearing et al., 2018). The common pooling of sequences into OTUs limited the possibilities of deep sequencing by preventing fine-scale resolution. Therefore, we chose to generate ASVs instead of OTUs to achieve state-of-the-art fine-scale community data and, in addition, to benefit from the error correction model applied by the DADA2 algorithm. In the following we will use the term *feature*, as introduced by QIIME2, when referring to the microbial ASVs (Boylen et al., 2018).

### Quantitative Real-Time PCR (qPCR)

For quantification of the domain-level specific primers (eubacterial and archaeal 16S rRNA genes) we followed the protocol from Bayer et al. (2014). Briefly, 1:5 dilutions of purified PCR products were used as standards, and all standard dilutions were prepared in aqueous tRNA solution (10 ng/ml) (Sigma-Aldrich, Schnelldorf, Germany). The DNA concentration of the highest starting solution of each standard dilution series as well as the diluted template DNAs was measured using the Qubit system (double stranded DNA, high sense kit, Thermo Fisher Scientific, Darmstadt, Germany). Quantitative PCRs were performed in a CFX Connect realtime detection system (Bio-Rad, Munich, Germany) using the SsoAdvanced^TM^ Universal SYBR^®^ Green Supermix (Bio-Rad) following the manufacturer’s instructions. For both primer pairs, the reaction conditions previously tested (see Bayer et al., 2014) were used with one exception: the annealing/elongation temperature for the archaeal 16S rDNA gene assay was increased to 66°C. For all qPCR assays, plate reads were taken at the end of each qPCR cycle. All template DNAs from sponges and seawater were tested in triplicates on each plate (technical replicates), whereas the corresponding standards were run in duplicates. The qPCR efficiency and gene copy numbers were calculated using the Bio-Rad CFX MANAGER^TM^ software (version 3.1). Amplification of specific targets was confirmed by analyses of melt curves (in steps of 0.5°C for 5 s, with temperatures ranging from 60 to 95°C). Additionally, PCR product sizes were checked by electrophoresis on a 1.5% agarose gel (Peqlab now VWR, part of avantor) in 1x TAE buffer with 0.5% GelGreen^TM^ (Biotium, Hayward, CA, United States) for visualization.

### Bacterial and Archaeal Community Analyses

Prokaryotic taxonomy tables from domain to species levels were created using the *qiime taxa barplot* plugin. A collective calculation of diversity metrics (both phylogenetic and non-phylogenetic) was applied on available datasets via the *qiime diversity core-metrics-phylogenetic* plugin, using the minimum sampling depth of each dataset for mandatory subsampling. The following alpha diversity indices were considered: Faith’s phylogenetic diversity, observed features, Shannon diversity, and evenness. Visualization of beta diversity used the principal coordinates results, which were based on weighted uniFrac distances. Analyses of sample composition in the context of categorical metadata were performed with the *qiime diversity beta-group-significance* plugin (parameters: *permanova* and *permdisp*) again utilizing the weighted uniFrac distances. The ggpubr R package was used to calculate sample statistics and plot respective results (i.e., alpha diversity and qPCR bar charts, or PCoA plots)^4^. Ternary plots were created using the Ternary plot maker web tool^5^. Finally, taxonomy bar plots and bacterial and archaeal features heatmaps were created using a custom R script and the ggplot2 package (Wickham, 2016). For heatmaps the respective feature abundance tables were sub-sampled for sponge samples only in order to avoid the bias introduced by abundant seawater features. The respective seawater samples were added subsequently before heatmap creation. Bacterial and archaeal feature Venn diagrams were created using mothur v.1.42.3 (Schloss et al., 2009). The complete QIIME2 pipeline and R script collection from the present study can be accessed online^6^.

### Archaeal Taxonomy Note

The applied SILVA 132 database places the family Nitrosopumilaceae within the phylum Thaumarchaeota (class Nitrososphaeria; order Nitrosopumilales). However, a recent prokaryotic taxonomical reassessment using full genomes reorganized the taxonomy of the family Nitrosopumilaceae considerably (i.e., phylum Crenarchaeota; class Nitrososphaeria; order Nitrososphaerales) (Parks et al., 2018). In terms of reproducibility we decided to use the available SILVA 132 taxonomy without manual changes to certain taxa, such as the family Nitrosopumilaceae. However, the presented taxonomy might be a subject to change in upcoming SILVA releases.

## Results

### Deep-Sea Sponge Taxonomy

Sponge taxonomic identifications were confirmed using a combination of gene markers, morphology, and spicule analyses. The 45 sponge specimens in this study belong to the two sponge classes Demospongiae and Hexactinellida. The Demospongiae group is composed of six taxa comprising the species *Geodia vaubani* Lévi and Lévi (1983), the novel species *Halichondria* sp. indet., *Latrunculia* sp. nov., the novel species *Paratimea* sp. indet., *Penares turmericolor* Sim-Smith and Kelly (2019), and *Pleroma turbinatum* Sollas (1888). The Hexactinellida group consists of seven taxa comprising the species *Aphrocallistes beatrix* Gray (1858), *Bolosoma cyanae* Tabachnick and Lévi (2004), *Corbitella plagiariorum* Reiswig and Kelly (2018), *Leucopsacus distantus* Tabachnick and Lévi (2004), *Regadrella okinoseana* Ijima (1896), *Saccocalyx tetractinus* Reiswig and Kelly (2018) and one novel species of Rossellidae that belongs to the Lanuginellinae subfamily (Table 1).

### Bacterial and Archaeal Features (Amplicon Sequencing)

The sequencing of sponge-associated prokaryotic communities and additional seawater samples yielded 9120 bacterial and 1052 archaeal features in total, whereas the sequencing depth was relatively balanced with 1,960,245 bacterial and 2,090,246 archaeal sequence reads (Table 2). Excluding seawater samples, Hexactinellida exhibited the most bacterial features (*n* = 4357), compared to the Demospongiae (*n* = 2084) specimens. In contrast, the demosponge samples exhibited a higher archaeal feature count (*n* = 437), compared to the hexactinellids (*n* = 264). Finally, the archaeal seawater feature count (*n* = 791) was higher in comparison with the two sponge classes, whereas bacterial seawater features were almost similar to the hexactinellid counts (*n* = 4025) (Table 2).

**TABLE 2:** Detailed bacterial and archaeal MiSeq library statistics, comprising the sequence data for (a) the whole dataset (i.e., Demospongiae, Hexactinellida, and seawater samples), (b) the Demospongiae subset, (c) the Hexactinellida subset, and (d) the seawater subset.

**Metric**	**Bacteria**	**Archaea**
Number of samples	72	72
Number of features	9,102	1,052
Total frequency	1,960,245	2,090,246
Demospongiae samples	21	21
Demospongiae features	2,084	437
Demospongiae frequency	558,729	770,140
Hexactinellida samples	24	24
Hexactinellida features	4,357	264
Hexactinellida frequency	588,341	535,764
Seawater samples	27	27
Seawater features	4,025	791
Seawater frequency	813,175	784,342

### Bacterial and Archaeal Taxonomy and Taxon Distribution

**FIGURE 2:**
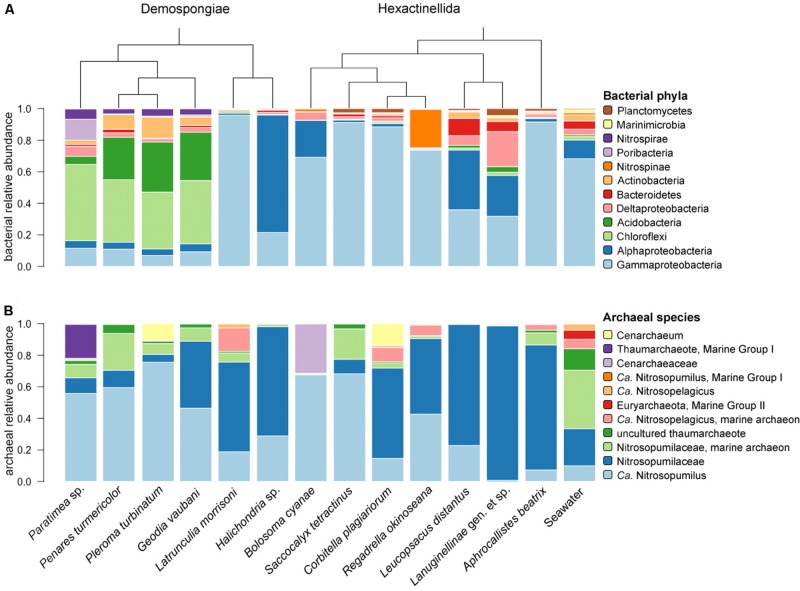
Relative abundance of the most abundant bacterial phyla **(A)** and archaeal species **(B)** for all 13 sponges and seawater. The dendrogram is based on phylogenetic relationships between the sponge species. Samples are grouped by sponge class (Demospongiae, Hexactinellida).

In total, 44 bacterial phyla and four archaeal phyla (i.e., Thaumarchaeota, Euryarchaeota, Nanoarchaeota, and Crenarchaeota) were present among all sponge and seawater samples. The demosponges *Paratimea* sp., *P. turmericolor*, *P. turbinatum*, and *G. vaubani*, contain Chloroflexi, Acidobacteria, Actinobacteria, and Nitrospirae as the most abundant bacterial phyla (Figure 2A). Surprisingly, the *Paratimea* sp. specimens possess a large fraction of poribacterial symbionts (Figure 2A). The Hexactinellida contain bacterial phyla, such as Proteobacteria (Delta-), Bacteroidetes, Nitrospinae, or Planctomycetes, which are relatively more abundant than in the investigated demosponge specimens (Figure 2A).

Due to the Thaumarchaeota dominance (92–100% relative abundance among all sample groups – see Supplementary Table S2) and the overall low archaeal feature richness (compared to the bacterial community – see Supplementary Figure S2), we considered the archaeal species composition as the relevant taxonomic level in our following community description throughout the present study. This is in contrast to the common phylum composition approach that has been used to describe sponge-associated bacterial communities. At species level a total of 76 archaeal taxa, compared to 1774 bacterial taxa, could be designated to all features present in the sponge and seawater samples. Among those 76 archaeal taxa the subset comprising the most abundant archaea was composed of almost only thaumarchaeotes, except one euryarchaeotal taxon (Thermoplasmata, Marine Group II) (Figure 2B). In addition, all top thaumarchaeotes belong to the family Nitrosopumilaceae (Figure 2B), which could be further identified as several *Candidatus* taxa, such as Nitrosopumilus or Nitrosopelagicus.

**FIGURE 3:**
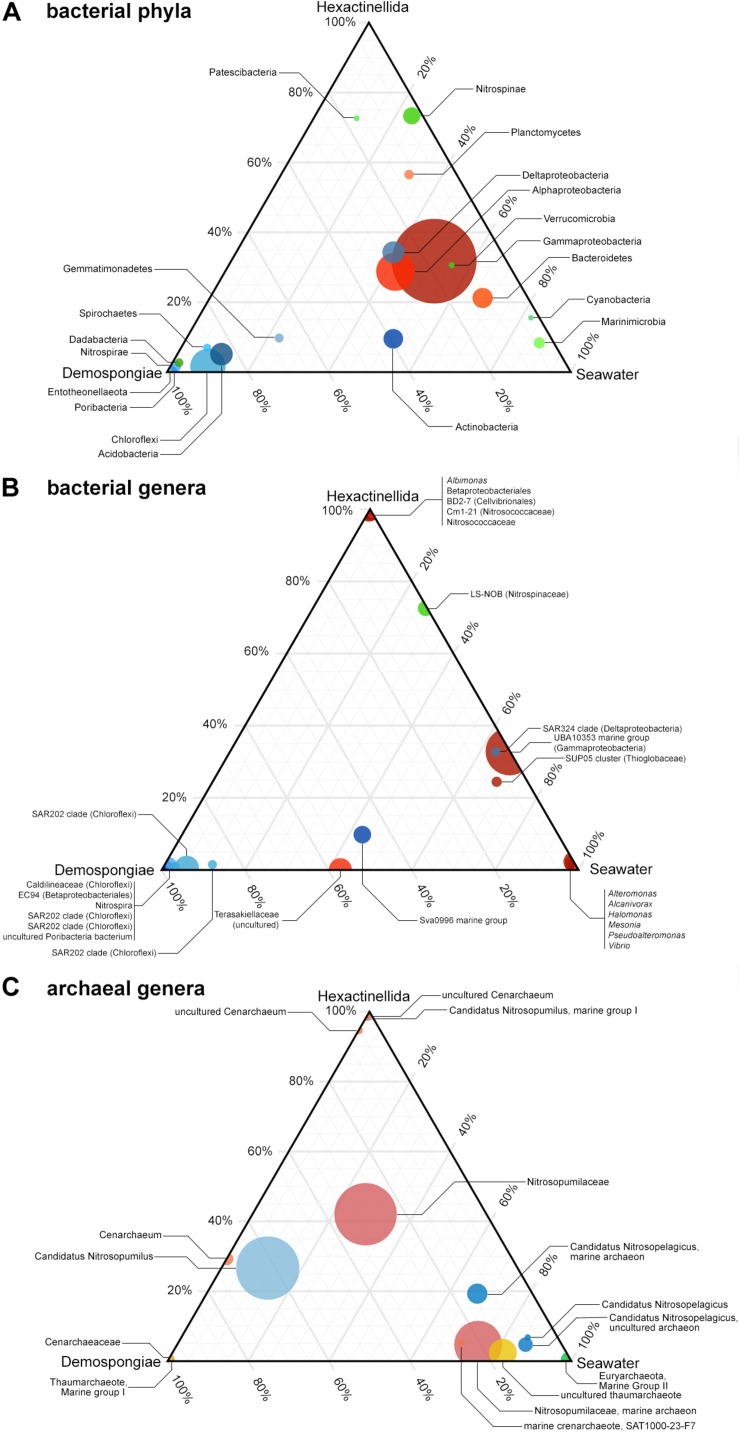
Ternary plots showing the distribution of the most abundant taxa of bacterial phyla **(A)**, bacterial genera **(B)**, and archaeal genera **(C)** among the pooled Demospongiae, Hexactinellida, and seawater samples. Circle size is indicative of the relative abundance of these phyla.

Ternary plots were used to examine the distributions of the bacterial and archaeal most abundant taxa among the pooled demosponges, hexactinellids, and seawater samples. As indicated by the ternary plots, certain bacterial phyla are almost exclusive to Demospongiae; such as Acidobacteria, Chloroflexi, Dadabacteria, Nitrospirae, Spirochaetes, Entotheonellaeota, or Poribacteria (Figure 3A). On the contrary, there is no evidence that the present hexactinellids possess an exclusive phylum. However, Patescibacteria and Nitrospinae seem to be more related to this sponge class as opposed to demosponges or the surrounding seawater. In addition, Marinimicrobia, Cyanobacteria, and Bacteroidetes exhibit a preference for the present seawater samples (Figure 3A). Finally, the group of several abundant proteobacterial classes, and Verrucomicrobia, show an almost even distribution among all three biotopes, however, with a clear tendency for the seawater samples.

On genus level there are some demosponge- (e.g., Caldilineaceae, EC94, SAR202 clade, and Poribacteria) or hexactinellid- (e.g., *Albimonas*, Betaproteobacteriales, Nitrosococcaceae, BD2-7, and Cm1-21) specific taxa that seem to be exclusive for one of the two sponge classes (Figure 3B). In addition, the seawater samples possess certain exclusive genera, such as *Alteromonas*, *Halomonas*, *Pseudoalteromonas*, or *Vibrio*.

Similar to the bacterial genera ternary plot, the most abundant archaeal assemblages can be grouped into either Demospongiae-, Hexactinellida-, or seawater-specific taxa. Here, certain taxa are highly specific to one of the available main groups (Figure 3C). For instance, the single euryarchaeotal taxon (Marine Group II) is only present in the seawater samples, whereas several Nitrosopumilaceae-related taxa are highly characteristic for one of the two sponge classes. Moreover, all three abundant *Candidatus* Nitrosopelagicus taxa have a strong preference for the seawater samples (Figure 3C). On the contrary, the *Candidatus* Nitrosopumilus taxa show either a tendency for demosponges and seawater samples or are unique to the Hexactinellida. Finally, a large group composed of not further classified Nitrosopumilaceae features is present almost at the center between all main biotopes, hinting to an even distribution of further Nitrosopumilaceae-related features among the three biotopes (Figure 3C).

### Alpha Diversity Analysis

Rarefied abundance tables, generated using the individual sample read counts and feature assemblages, were used to calculate the mean bacterial and archaeal feature diversity at a local scale (i.e., alpha diversity indices for demosponges, hexactinellids, and seawater groups). Alpha diversity analyses of the bacterial communities revealed that all four indices (i.e., Faith’s PD, observed features, Shannon diversity, and evenness) were significantly different within both sponge classes (Table 3). In contrast, the archaeal feature assemblages showed no significant alpha diversity differences between the species belonging to the two sponge classes. Seawater samples apparently deviated from that overall pattern by exhibiting almost no significant differences for the two present richness indices, except the observed archaeal features (*p* = 0.044), whereas Shannon diversity and evenness showed significant differences among the seawater samples for both prokaryotic domains (Table 3). In addition, the comparisons between the three biotopes showed significant differences among all available alpha diversity indices for bacteria and archaea (Supplementary Figure S2). However, significant *p*-values were lower for the archaeal features compared to the bacterial *p*-values. Moreover, Faith’s PD and observed feature values for bacteria and archaea were highest within the seawater samples, followed by demosponges and hexactinellids (Supplementary Figure S2). The same pattern was visible in the bacterial and archaeal Shannon and evenness indices, where the spread was much higher in the sponge related samples compared to the richness indices (Supplementary Figure S2).

**TABLE 3:** Bacterial and Archaeal alpha diversity for Demonspongiae, Hexactinellida, and seawater.

		**Demospongiae**		**Hexactinellida**		**Seawater**	
		**H**	***p*-value**	**H**	***p*-value**	**H**	***p*-value**
**Faith’s PD**	**Bacteria**	14.37	**0.013**	18.17	**0.006**	15.33	0.053
	**Archaea**	6.09	0.298	4.50	0.480	11.93	0.155
**Features**	**Bacteria**	18.07	**0.003**	18.14	**0.006**	14.10	0.079
	**Archaea**	5.43	0.365	4.00	0.677	15.90	**0.044**
**Shannon**	**Bacteria**	18.97	**0.002**	18.67	**0.005**	19.96	**0.010**
	**Archaea**	3.71	0.592	6.29	0.391	21.19	**0.007**
**Evenness**	**Bacteria**	18.48	**0.002**	19.11	**0.004**	19.15	**0.014**
	**Archaea**	4.22	0.518	6.57	0.363	17.40	**0.026**

*Significant *p* values are highlighted in bold.*

### Beta Diversity Analysis

**FIGURE 4:**
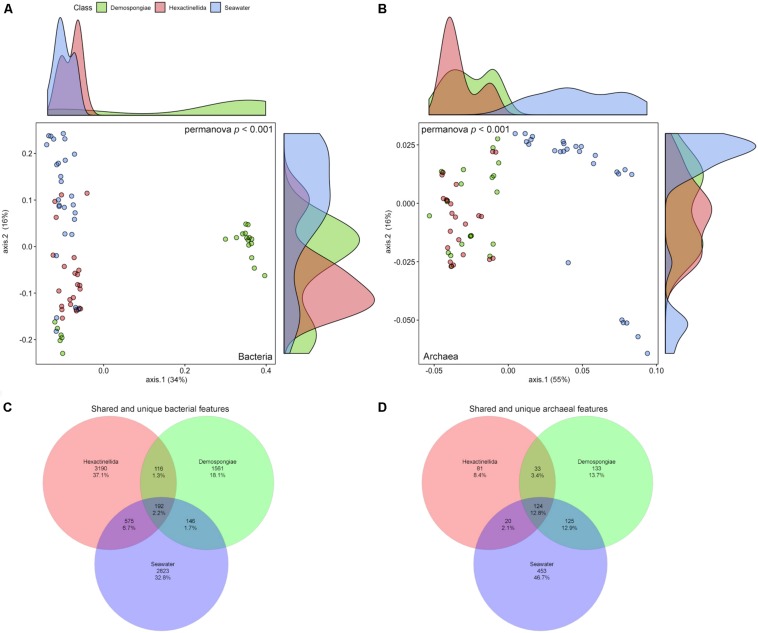
Principal component analysis using bacterial **(A)** and archaeal **(B)** weighted uniFrac distances for Demospongiae, Hexactinellida and seawater samples. Venn diagrams depicting the shared and unique bacterial **(C)** and archaeal **(D)** features as percent for the three sampling groups (Hexactinellida, Demospongiae, seawater).

The same rarefied abundance tables that were used in the alpha diversity analyses were again utilized to look at several beta diversity aspects (i.e., community differences between samples). First, the bacterial and archaeal abundance and composition information was employed to investigate the differences between the Demospongiae, Hexactinellida, and seawater samples (Figures 4A,B). Regarding the bacterial community, two main groups are visibly separated by the first axis (34% variance explained) (Figure 4A). The larger group is composed of all Hexactinellida, seawater, and a Demospongiae subset, whereas the second group exclusively consists of demosponges. However, the larger group also exhibits a relatively distinct separation between the seawater, Hexactinellida, and Demospongiae samples. In this group, the second axis separates the seawater- and Demospongiae subsets, while the Hexactinellida prevailing subsets are in-between these two distinct groups (16% variance explained). Nevertheless, a few overlaps between these biotopes exist (Figure 4A). Comparative statistics showed that the group identity has a significant effect on the community composition (*p* < 0.001), while the variances were homogeneous (*p* = 0.083) (Figure 4A and Table 4).

**TABLE 4:** Community permanova and permdisp statistics, comprising the sequence data for (a) the whole dataset (i.e., Demospongiae, Hexactinellida, and seawater samples), (b) the Demospongiae subset, (c) the Hexactinellida subset, and (d) the seawater subset.

	**Group**	**Sample size**	**Groups**	**Test statistic**	***p*-value**
**Bacteria**	Group permanova	72	3	15.27	**0.001**
	Group permdisp	72	3	2.53	0.083
	Demo permanova	21	6	40.99	**0.001**
	Demo permdisp	21	6	30.09	**0.013**
	Hexac permanova	24	7	7.74	**0.001**
	Hexac permdisp	24	7	4.50	**0.002**
	SW permanova	27	9	7.35	**0.001**
	SW permdisp	27	9	0.58	0.451
**Archaea**	Group permanova	72	3	32.52	**0.001**
	Group permdisp	72	3	0.09	0.895
	Demo permanova	21	6	1.22	0.203
	Demo permdisp	21	6	1.82	0.19
	Hexac permanova	24	7	1.28	0.106
	Hexac permdisp	24	7	0.56	0.496
	SW permanova	27	9	20.99	**0.001**
	SW permdisp	27	9	0.47	0.235

*Significant *p* values are highlighted in bold.*

In case of the archaeal feature composition and abundance, the first axis clearly separates the sponge samples from the seawater samples (55% variance explained) (Figure 4B). Furthermore, seawater samples are then subset by the second axis (16% variance explained) into one large and one small group. The smaller group is comprised of seawater samples from ROV station 13 (Otago-Canterbury slope) and station 14 (Christchurch slope), which are both located on the continental slope of the South Island within the subtropical front separating the water masses of the South Pacific Subtropical Gyre to the north from the Subantarctic Water Ring to the south (Carter, 2001; Figure 1 and Supplementary Figure S3, Table 1). In comparison, the sponge samples exhibit no clear separation into Demospongiae and Hexactinellida specimens, hence forming a heterogeneous group apart from the seawater samples (Figure 4B). However, overall the archaeal composition was still significantly affected by group identity (*p* < 0.001), further supported by homogeneously dispersed groups (*p* = 0.895) (Figure 4B and Table 4).

Finally, biotope-specific subsets (i.e., Demospongiae, Hexactinellida, and seawater) revealed that the demosponge and/or hexactinellid host-identity has a significant effect on the bacterial community composition (*p* < 0.001 for both sponge classes) (Supplementary Figures S3A,C and Table 4), whereas no significant effect of sponge identity could be observed for the archaeal data (*p* = 0.203 and *p* = 0.106 for demosponges and hexactinellids, respectively) (Supplementary Figures S3B,D and Table 4). In comparison, sampling location has a significant effect on the bacterial and archaeal community composition of the seawater samples (Supplementary Figures S3E,F and Table 4).

### Bacterial and Archaeal Feature Distribution and Abundance

Venn diagrams visualized the shared and unique bacterial and archaeal features for the three sampling groups (Figures 4C,D). Overall, the percentage of shared bacterial features among all three groups is low and consequently each of the sample groups exhibits a large share of unique features (37.1, 18.1, and 32.8% for Hexactinellida, Demospongiae, and seawater, respectively) (Figure 4C). Similarly, for the archaeal features, the pooled seawater samples hold the largest share of the archaeal features (46.7%) compared to the demosponges (13.7%) and hexactinellids (8.4%) (Figure 4D). Moreover, some shared features (i.e., 12.9% for seawater/Demospongiae, and 12.8% for seawater/Demospongiae/Hexactinellida) exceed the Hexactinellida-unique features. Despite the large fraction of seawater-unique features the amount of shared features between Hexactinellida and seawater is low (2.1%).

**FIGURE 5:**
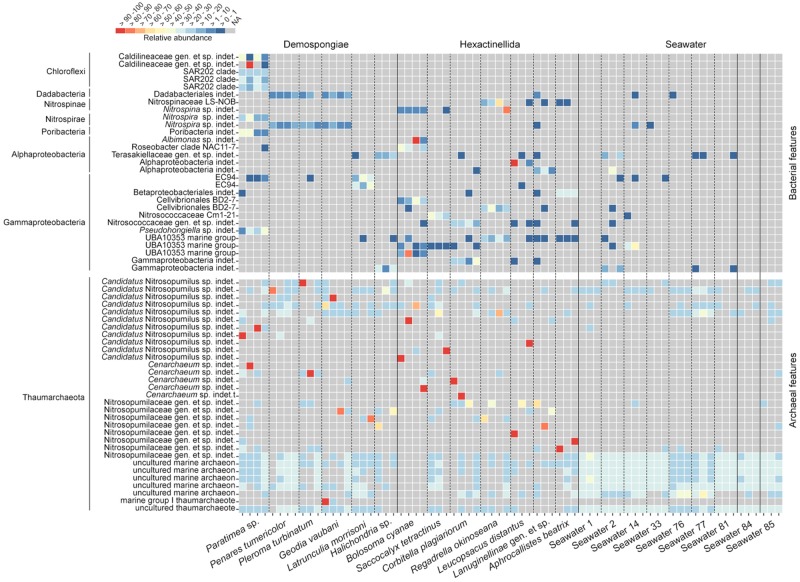
Relative abundance heatmap of the investigated Demospongiae, Hexactinellida, and seawater. Heatmaps are separated between bacterial and archaeal features. Lowest available classification per feature has been used to assign individual taxonomy. Features were subsequently sorted by phylum.

We plotted the most abundant bacterial and archaeal features within the sponge subset as separated relative abundance heatmaps (Figure 5). The bacterial heatmap reveals that certain features are either highly host-species specific (e.g., Caldilineaceae gen. et sp. indet., SAR202 clade, *Nitrospira* sp., *Pseudohongiella* sp., *Nitrospina* sp., Roseobacter clade NAC11-7, EC94, Cellvibrionales BD2-7, or Nitrosococcaceae) or predominant in the respective sponge classes (Demospongiae: Dadabacteriales, *Nitrospira* sp.; Hexactinellida: UBA10353 marine group). Overall, the distribution among the hexactinellid samples appears to be more scattered compared to the demosponge samples. Finally, seawater does not possess any of the sponge-specific bacterial features in large abundances and across all samples from the same location (Figure 5). The archaeal heatmap revealed that individual sponges possess single highly abundant features, which all belong to different Nitrosopumilaceae taxa (Figure 5). Contrary to the host-species specific distribution of the bacteria, sponge-associated archaeal symbionts are apparently individual-specific rather than host-specific. In contrast, seawater samples do not possess these singular highly abundant features. Instead, those samples rather exhibit location-specific feature distribution patterns (Figure 5). This corresponds with the archaeal ordination plots (Figure 4 and Supplementary Figure S3) and the respective archaeal community statistics (Table 4). Concerning the sponge-associated archaeal features, there seems to be a tendency that those singular abundant archaea predominantly belong to certain taxa (i.e., *Candidatus* Nitrosopumilus or Cenarchaeum).

### Quantitative Assignment of Sponge-Associated Bacterial and Archaeal 16S rRNA Genes

**FIGURE 6:**
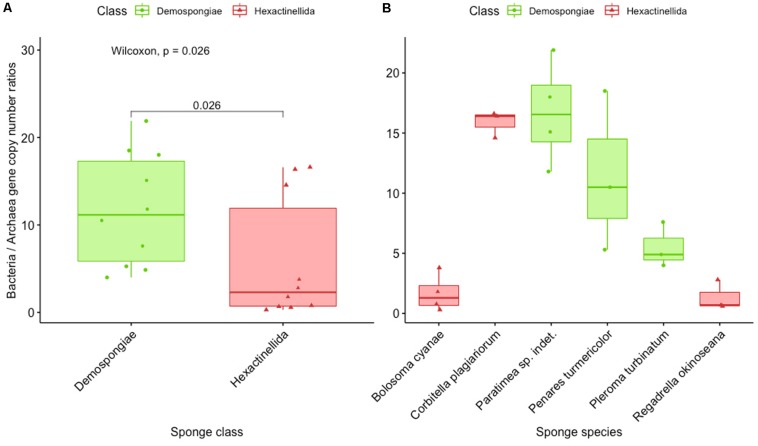
Results of the qPCR experiments for bacterial and archaeal abundance presented as ratio of bacteria and archaea gene copy numbers. **(A)** Representing the overall average for Demospongiae and Hexactinellida. **(B)** Showing the ratio of bacteria and archaea gene copy numbers for three species of Demospongiae and Hexactinellida, respectively.

For both qPCR assays the published reaction conditions (Bayer et al., 2014) were re-tested with respect to primer concentration, annealing, and elongation temperature and time. Both assays showed good *in silico* coverage and specificity and showed qPCR efficiencies between 95 and 105% with external standards according to MIQE guidelines (Bustin et al., 2009). One exception was that the annealing/elongation temperature for the archaeal assay was increased to 66°C for specificity reasons but without reaction efficiencies loss (Supplementary Table S3). All qPCR results are expressed as gene copy numbers per microgram genomic DNA. The 16S rRNA gene copy numbers as estimated by qPCR with domain-specific primers for the investigated sponges are reported as follows (Supplementary Table S4). Bacterial 16S rRNA gene copy numbers ranged from 5.05 × 10^10^ ± 1.54 × 10^10^ to 2.53 × 10^11^ ± 2.55 × 10^10^ in hexactinellid sponges, to 5.62 × 10^10^ ± 4.79 × 10^9^ to 1.81 × 10^11^ ± 3.46 × 10^10^ in Demospongiae. Archaeal 16S rRNA gene copy numbers varied from 2.01 × 10^9^ ± 1.23 × 10^8^ to 2.71 × 10^11^ ± 2.57 × 10^10^ in hexactinellid sponges, to 3.09 × 10^8^ ± 1.29 × 10^7^ to 3.24 × 10^10^ ± 1.73 × 10^9^ in Demospongiae (Supplementary Figure S4 and Supplementary Table S4). As the fraction of host DNA within total extracted DNA potentially varies in every sample, we further calculated the ratios of bacteria and archaeal 16S rRNA gene copy numbers. We found that the prokaryotic consortia of hexactinellid sponges *B. cyanae* and *R. okinoseana* seem to have a high proportion of associated Archaea (BAC: ARCH ratios between 0.3 and 3.8), whereas *C. plagiariorum* deviates from that (BAC: ARCH ratios between 14.6 and 16.6). Demospongiae samples showed a higher proportion of Bacteria on average compared to Archaea (BAC: ARCH ratios between 4.0 and 21.9), with one apparent exception, which is *L. morrisoni* (BAC: ARCH ratios between 171.1 and 322.1) (Supplementary Table S4). Finally, the conversion of 16S rRNA gene copy numbers into BAC: ARCH ratios showed that Archaea, compared to the bacterial copy numbers, are significantly (*p* = 0.026) more abundant in hexactinellids than in demosponges (Figure 6A). However, a closer look at the individual sponge species revealed one exception from that initial observation (Figure 6B; *C. plagiariorum* with a BAC: ARC ratio between 14.6 and 16.6). A potential limitation of using the bacteria vs. archaeal ratio could be a possible bias in primer amplification, because of the use of two different 16S rRNA gene primer pairs.

## Discussion

### Bacterial Communities in Deep-Sea Demosponges and Hexactinellids

Since the beginning of sponge microbiome research (Vacelet, 1975; Vacelet and Donadey, 1977) the emphasis was placed on sponges from shallow-water marine habitats while sponges from cold-water and/or deep-sea locations are still understudied. Here, we explored the microbial community composition of demosponges and hexactinellids from the South Pacific by constructing independent bacterial and archaeal partial 16S rRNA gene libraries. While collection of sponges during the SO254 expedition included only deep-water specimens, analyses of microbiota patterns between deep-water and shallow-water sponges relied on literature data for shallow-water microbiota data (e.g., Thomas et al., 2016; Moitinho-Silva et al., 2017b).

The six demosponges showed a microbial signature similar to that of shallow-water demosponges with the phyla Proteobacteria (Gamma-, Alpha-, and Delta-), Chloroflexi, Acidobacteria, Bacteroidetes, Nitrospinae, Nitrospirae, and Poribacteria being most abundant. One apparent difference to shallow-water sponges is the general lack or low abundance of members of the phylum Cyanobacteria, which are typically more abundant in shallow-water sponges (e.g., Erwin and Thacker, 2008; Bayer et al., 2014; Burgsdorf et al., 2014; Thomas et al., 2016). Given their involvement in photosynthesis it is not surprising that the present deep-sea sponges contain reduced numbers of this phototrophic bacterial phylum. The highly sponge-specific phylum Poribacteria appears to be underrepresented in sponges from deep-sea or cold habitats in the present and previous studies (Jackson et al., 2013; Rodríguez-Marconi et al., 2015; Cárdenas et al., 2018; Steinert et al., 2019). Noteworthy, we identified one sponge species (*Paratimea* sp.) with abundant poribacterial features. The overall phylum composition of this sponge matches the typical taxon composition of high microbial abundance (HMA) sponges as defined by Moitinho-Silva et al. (2017b). Also, three more demosponges in this study (i.e., *P. turmericolor*, *P. turbinatum*, and *G. vaubani*) exhibited these HMA indicator taxa, although Poribacteria were lacking. The two remaining demosponge species (i.e., *Latrunculia* sp. nov. and *Halichondria* sp. indet.), display microbiome characteristics typical of LMA sponges (see Figure 2). In summary, with respect to bacterial community composition and HMA/LMA status, the demosponges from this remote deep-sea location largely resemble those of shallow-water collections (Thomas et al., 2016).

Regarding the seven hexactinellid species in the present study there is no literature available for comparisons with other hexactinellids from similar or different habitats. Overall, Gammaproteobacteria dominated the hexactinellid species. While Gammaproteobacteria are common predominant members in sponge bacterial communities, usually those demosponge-related communities exhibit additional dominant taxa regardless of climate zone or sampling depth (e.g., Kennedy et al., 2014; Thomas et al., 2016; Cárdenas et al., 2018; Steinert et al., 2019). Two species, i.e., Lanuginellinae gen. et sp. and *L. distantus*, also possess abundant Proteobacteria (Alpha- and Delta-), Bacteroidetes, and Chloroflexi, thus resembling a bacterial community pattern commonly found in demosponges.

When comparing the two sponge classes, evenness and diversity were indeed lower in hexactinellids compared to demosponges. Besides these alpha-diversity metrics, bacterial community composition and diversity differences are prominent throughout all present analyses. For instance, at least some demosponges possess specific phyla, like the HMA indicators, whereas hexactinellids resemble LMA sponges regarding their bacterial taxon composition. Secondly, demosponges and hexactinellids do not overlap in the ordination analyses, hence hexactinellids possess a rather class-specific microbiota. This is especially apparent when comparing the feature distribution between LMA-like demosponges and hexactinellids. The Hexactinellida-associated features exhibit a more heterogeneous distribution. Hexactinellida are usually cold water/deep-sea sponges (van Soest et al., 2012). Such habitats imply lower food availability and therefore different metabolic functions in species of this sponge class. Moreover, glass sponges are clearly distinct to other sponge classes by body shape and features such as tissue and spicules (van Soest et al., 2012). We assume that these morphological differences also affect the microbiota composition of this class. Sponges are holobionts (Webster and Thomas, 2016); hence the symbiotic bacterial relationships are adapted to the Pita et al. (2018) host-ecosystem, which could be visible as discernable differences between demosponges and hexactinellid bacterial communities.

Demospongiae and Hexactinellida also share some common sponge-bacteria related characteristics, such as their noticeable difference to seawater samples (e.g., Lee et al., 2010; Taylor et al., 2013; Steinert et al., 2016; Cleary et al., 2018; Helber et al., 2019), which is present in the ordination plots or manifested in the alpha- and beta-diversity results. Especially apparent is the Hexactinellida-related host-specificity (see Supplementary Figure S3C), which is equally consistent in their demosponge counterparts (see Supplementary Figure S3A). Host-specificity is a common feature of the sponge-microbiota relationship, but so far only observed and described in depth for demosponges (Pita et al., 2013; Easson and Thacker, 2014; Thomas et al., 2016; Steinert et al., 2017, 2019). This pattern seems to be similar in hexactinellids, implying analogous bacterial community acquisition and maintenance processes as in their demosponge counterparts.

### Archaeal Communities in Deep-Sea Demosponges and Hexactinellids

At high taxonomic archaeal ranks (i.e., from phylum to family level), both Demospongiae and Hexactinellida are dominated or even exclusively inhabited by the phylum Thaumarchaeota, and more specifically, several genera and species from the family Nitrosopumilaceae. The ecologically important *candidatus* family Nitrosopumilaceae forms a monophyletic group in the *candidatus* order Nitrosopumilales based on 16S rRNA and *candidatus amoA* (encoding for the α-subunit of ammonia monooxygenase) gene sequence analyses (Torre et al., 2016). The Nitrosopumilaceae comprises five genera, three of which were present in our sponges, namely: *Candidatus* Nitrosopelagicus, *Candidatus* Nitrosopumilus, and *Candidatus* Cenarchaeum. Nitrosopumilaceae grow chemolithoautotrophically by acquiring energy from ammonia oxidation and using CO_2_ as carbon source. Additionally, some species can utilize urea as a source of ammonia for energy and growth (Torre et al., 2016). Marine demosponges are known hosts of symbiotic thaumarchaeotal members, but the understanding of their functional relationship is still lacking and mostly relies on circumstantial evidence (e.g., Kennedy et al., 2014; Feng et al., 2016, 2018; That et al., 2018; Moeller et al., 2019).

Different nitrogen cycling processes, such as nitrification, denitrification, and anaerobic ammonium oxidation have been observed in different demosponge species (e.g., Bayer et al., 2008; Hoffmann et al., 2009; Schläppy et al., 2010; Radax et al., 2012). Ammonia-oxidizing archaea (AOA) (i.e., thaumarchaeotes) are often abundant and diverse members of the sponge microbiota. AOA have even been detected in sponge larvae indicating vertical transmission and/or early environmental acquisition (Sharp et al., 2007; Steger et al., 2008; Schmitt et al., 2012). Transcription and translation of important functional genes, like the *amoA* gene of thaumarchaeotes, has been observed in different demosponge species (e.g., Bayer et al., 2008; Liu et al., 2012; Radax et al., 2012; Moitinho-Silva et al., 2017a). More complete genomic information about members of the phylum Thaumarchaeota, or more specifically the family Nitrosopumilaceae, has shed light on the metabolic potential and functional relationships of this putatively important sponge-symbiotic archaeal group. So far, genomic information comprising different taxa within the family Nitrosopumilaceae is available from three demosponges (*Axinella mexicana*, *Cymbastela concentrica*, *Ianthella basta*), and even one hexactinellid (*Lophophysema eversa*) (Hallam et al., 2006; Tian et al., 2016; Moitinho-Silva et al., 2017a; Moeller et al., 2019). Given the involvement of the Nitrosopumilaceae family in sponge-related nitrogen cycling processes, it can be assumed that thaumarchaeotal ammonia oxidation is a key functional process in deep-sea demosponges and hexactinellids alike.

### Nitrosopumilaceae Are Sponge- but Not Sponge Species-Specific

We performed a high resolution screening of prokaryotic communities in demosponges and hexactinellids to look at the broad archaeal community in greater detail, and to directly compare community patterns and absolute abundances between bacterial and archaeal domains. Therefore, we constructed two independent bacterial and archaeal 16S rRNA gene libraries and quantitative real-time PCR in this study.

The difference between bacterial and archaeal communities is most apparent in sponge host-specificity, either at sponge class rank and/or at sponge species level (Figures 4A,B and Supplementary Figures S4A,B). Generally, bacterial demosponge-associated communities exhibit stable host-species specific patterns, which are affected by sponge-host identity and even sponge phylogeny (Easson and Thacker, 2014; Thomas et al., 2016; Steinert et al., 2017). The present archaeal communities do not show any distinct host-relatedness among all sponge species in both classes. Another difference between bacterial and archaeal community relationships is the feature distribution. Unique Nitrosopumilaceae features are randomly distributed among individual specimens and sponge taxa (Figure 5). In contrast, dominant sponge-associated bacteria often displayed sponge species-specific distribution patterns that are generally explained by particular metabolically and functional host–symbiont relationships (Fan et al., 2012; Ribes et al., 2012; Thomas et al., 2016). The present archaeal feature distribution indicates that a random symbiont acquisition process is present among all sponges, which is independent of host phylogeny. Nevertheless, we assume that the latter processes are sponge-specific due to the evident differences between sponge- and seawater-associated archaeal communities (Figures 4B, 5).

In summary, Nitrosopumilaceae acquisition seems to happen randomly across all sponge samples. This implies functional redundancy of the symbiont’s key functional processes within the deep-sea sponge-associated genera of the family Nitrosopumilaceae. Functional redundancy, or evolutionary equivalence, has been hypothesized and observed also for sponge-associated bacteria, but usually at higher taxonomic ranks (Fan et al., 2012; Ribes et al., 2012; Thomas et al., 2016).

### Bacterial and Archaeal Abundance

Finally, quantitative real-time PCR using both bacterial and archaeal universal 16S rRNA gene primers support the overall results. Although qPCR is a very sensitive method to assess microbial quantities, there are technical aspects that need to be considered. For example, the amplification of free DNA in sea water and sponge samples cannot be excluded and numbers might be overestimated. One explanation for the relatively high values could be the detection of dead material deposits from the upper water column in all three biotopes (i.e., Demospongiae, Hexactinellida, and seawater). As environmental microbes may encode for more than one 16S rRNA gene per genome, we express microbial abundances as gene copy number per microgram genomic DNA and compare it to literature (Bayer et al., 2014). Bacterial gene copy numbers were within the range found in shallow-water HMA demosponges (Bayer et al., 2014) and no significant difference was found comparing demosponges and hexactinellids from the deep sea (this study). Archaeal gene copy numbers detected in both deep-sea demosponges and hexactinellids were up to three orders of magnitude higher than in shallow-water counterparts (see Bayer et al., 2014), highlighting the importance of archaeal symbionts for deep-sea sponges in general. Since cold water carries more CO_2_ than warmer water (Garrison, 2009) the physiological evidence for archaea using CO_2_ as a carbon source (Wuchter et al., 2004; Könneke et al., 2014) might explain their higher abundance in deep-sea waters and sponges where ammonium for nitrification purposes might not be limited. Altogether, this suggests a major role of AOA in deep-sea sponge metabolism (in particular for the hexactinellid species), by providing additional metabolites via chemoautotrophy, similar to what diatoms or cyanobacteria contribute via photoautotrophy in shallow-water sponges (see Feng and Li, 2019 and references within). Hence, future (meta)-omic’s studies should explore if deep-water sponge microbiomes also contain chemoautotrophic bacteria, which could provide further resources to the host sponges in addition to the AOA.

## Conclusion

We investigated bacterial and archaeal communities from two sponge classes using independent 16S rRNA gene libraries and quantitative real-time PCR. With regard to bacteria, both demosponges and hexactinellids exhibit community characteristics similar to shallow-water sponges, including the presence of typical sponge symbiont taxa as well as host species-specific microbiomes for all sponge species investigated. In contrast, the archaeal community was taxonomically highly homogeneous and could only be resolved from the Nitrosopumilaceae family level on downward (i.e., from family to individual ASVs). However, the quantitative information hints at three orders higher archaeal gene copy numbers between shallow water and the present deep-water sponges. Hence, it is apparent that AOA are important members of deep-sea sponges in particular, and that different acquisition and maintenance processes may be involved regarding the archaeal symbionts compared to the bacteria.

## Data Availability Statement

Sequences were deposited at NCBI as BioProjects with accession IDs PRJNA552490 and PRJNA552540.

## Author Contributions

PS, UH, KaB, and GS designed the experiments. GS, KaB, KrB, GW, PS, SK, and PA performed the experiments. GS, DE, KaB, KrB, UH, and PS analyzed the data. GS, KaB, KrB, PS, and UH wrote the manuscript. GS, GW, KaB, KrB, MD, SM, UH, and PS reviewed and edited the manuscript. MD identified hexactinellid specimens. MK identified demosponge specimens. SM provided initial identification and curation of taxonomic vouchers of sponges.

## Conflict of Interest

The authors declare that the research was conducted in the absence of any commercial or financial relationships that could be construed as a potential conflict of interest. The reviewer NV declared several past co-authorship with the authors DE and GW to the handling editor.

## Footnotes

1 http://jlsrf.org/index.php/lsf/article/view/160

2 https://docs.qiime2.org/2018.11/tutorials/feature-classifier/

3 https://www.arb-silva.de/download/archive/qiime

4 https://cran.r-project.org/web/packages/ggpubr/

5 https://www.ternaryplot.com/

6 https://github.com/marinemoleco/So254Qiime2ASVs

## Supplementary Material

FIGURE S1 Pictures of the freshly collected sponges, including one picture of the whole specimen and one close-up picture. Demospongiae: **(A,B)** = *Pleroma turbinatum* Sollas, 1888; **(C,D)** = *Latrunculia* sp. nov.; **(E,F)** = *Paratimea* sp. indet; **(G,H)** = *Penares turmericolor* Sim-Smith and Kelly, 2019; **(I,J)** = *Geodia vaubani* Lévi and Lévi, 1983; **(K,L)** = *Halichondria* sp. indet. Hexactinellida: **(A,B)** = *Aphrocallistes beatrix* Gray, 1858; **(C,D)** = *Bolosoma cyanae* Tabachnick and Lévi, 2004; **(E,F)** = *Corbitella plagiariorum* Reiswig and Kelly, 2018; **(G,H)** = Lanuginellinae gen. et sp. indet; **(I,J)** = *Leucopsacus distantus* Tabachnick and Lévi, 2004; **(K,L)** = *Regadrella okinoseana* Ijima, 1896; **(M,N)** = *Saccocalyx tetractinus* Reiswig and Kelly, 2018.

FIGURE S2 Alpha diversity plots of bacterial and archaeal communities. Following indices are shown: **(A,B)** = Faith’s PD; **(C,D)** = observed features; **(E,F)** = Shannon diversity; **(G,H)** = Pielou’s evenness.

FIGURE S3 Principal component analysis using bacterial and archaeal weighted uniFrac distances for Demospongiae **(A,B)**, Hexactinellida **(C,D)**, and seawater **(E,F)** samples.

FIGURE S4 16S rRNA gene copy numbers of **(A)** bacterial and **(B)** archaeal quantitative real-time PCR data.

TABLE S1 Detailed sampling list, including internal sampling IDs, ROV site IDs, coordinates, and list of seawater samples.

TABLE S2 Archaeal relative abundances at phylum level.

TABLE S3 Quantitative real-time PCR efficiency values.

TABLE S4 Quantitative real-time PCR raw results. Including Qubit measurements, eubacterial 16S rRNA gene copy numbers, eubacterial 16S rRNA gene copy numbers/μg, and ratios.
