# Supplementary figures and images for: Compositional and Quantitative Insights Into Bacterial and Archaeal Communities of South Pacific Deep-Sea Sponges (Demospongiae and Hexactinellida)
Source: Front Microbiol. 2020 Apr 24;11:716. doi: 10.3389/fmicb.2020.00716 (PMC7193145; doi:10.3389/fmicb.2020.00716)

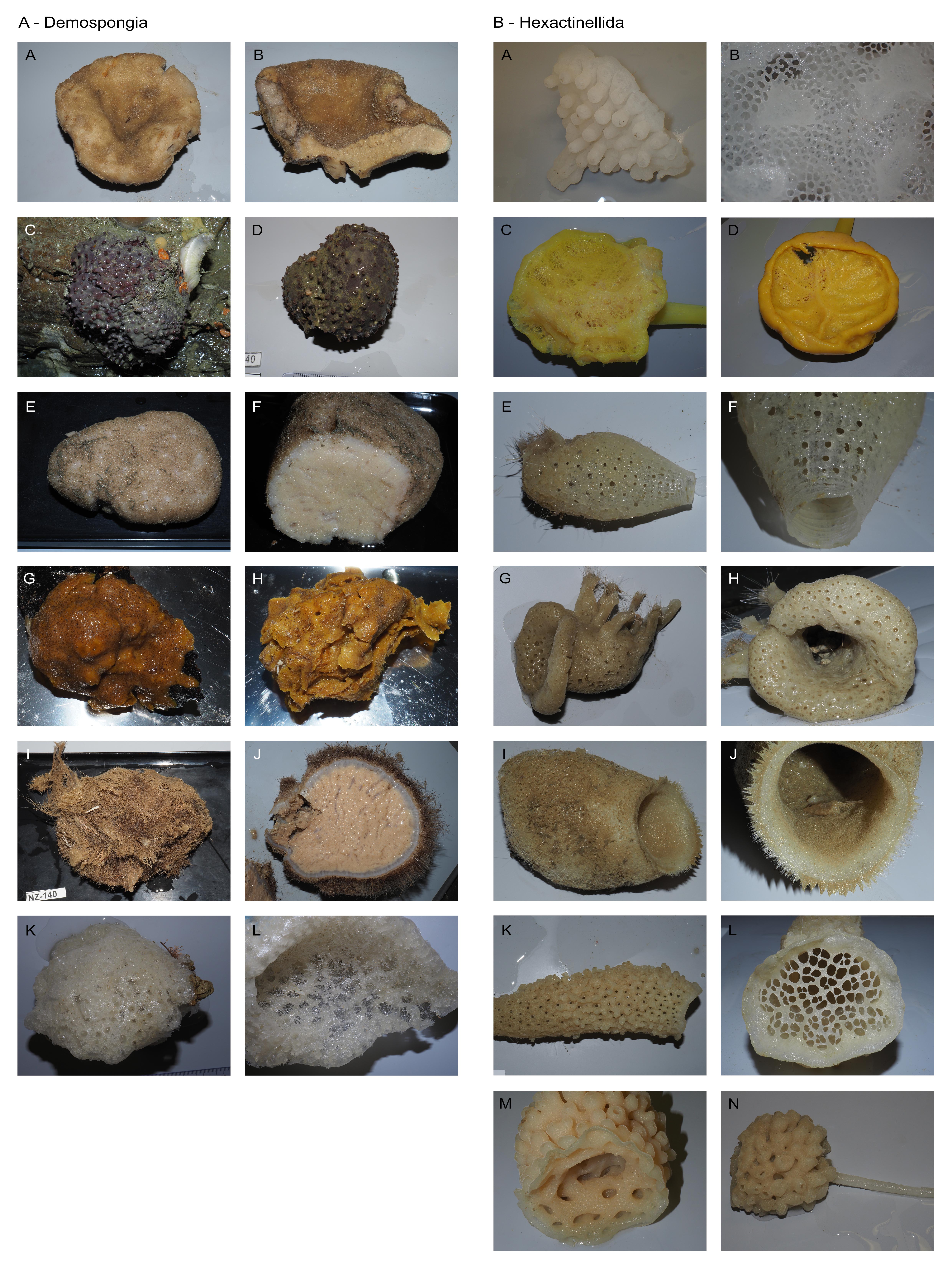

Supplement: FIGURE S1 — Pictures of the freshly collected sponges, including one picture of the whole specimen and one close-up picture. Demospongiae: (A,B) = Pleroma turbinatum Sollas, 1888; (C,D) = Latrunculia sp. nov.; (E,F) = Paratimea sp. indet; (G,H) = Penares turmericolor Sim-Smith and Kelly, 2019; (I,J) = Geodia vaubani Lévi and Lévi, 1983; (K,L) = Halichondria sp. indet. Hexactinellida: (A,B) = Aphrocallistes beatrix Gray, 1858; (C,D) = Bolosoma cyanae Tabachnick and Lévi, 2004; (E,F) = Corbitella plagiariorum Reiswig and Kelly, 2018; (G,H) = Lanuginellinae gen. et sp. indet; (I,J) = Leucopsacus distantus Tabachnick and Lévi, 2004; (K,L) = Regadrella okinoseana Ijima, 1896; (M,N) = Saccocalyx tetractinus Reiswig and Kelly, 2018. [file Image_1.JPEG]

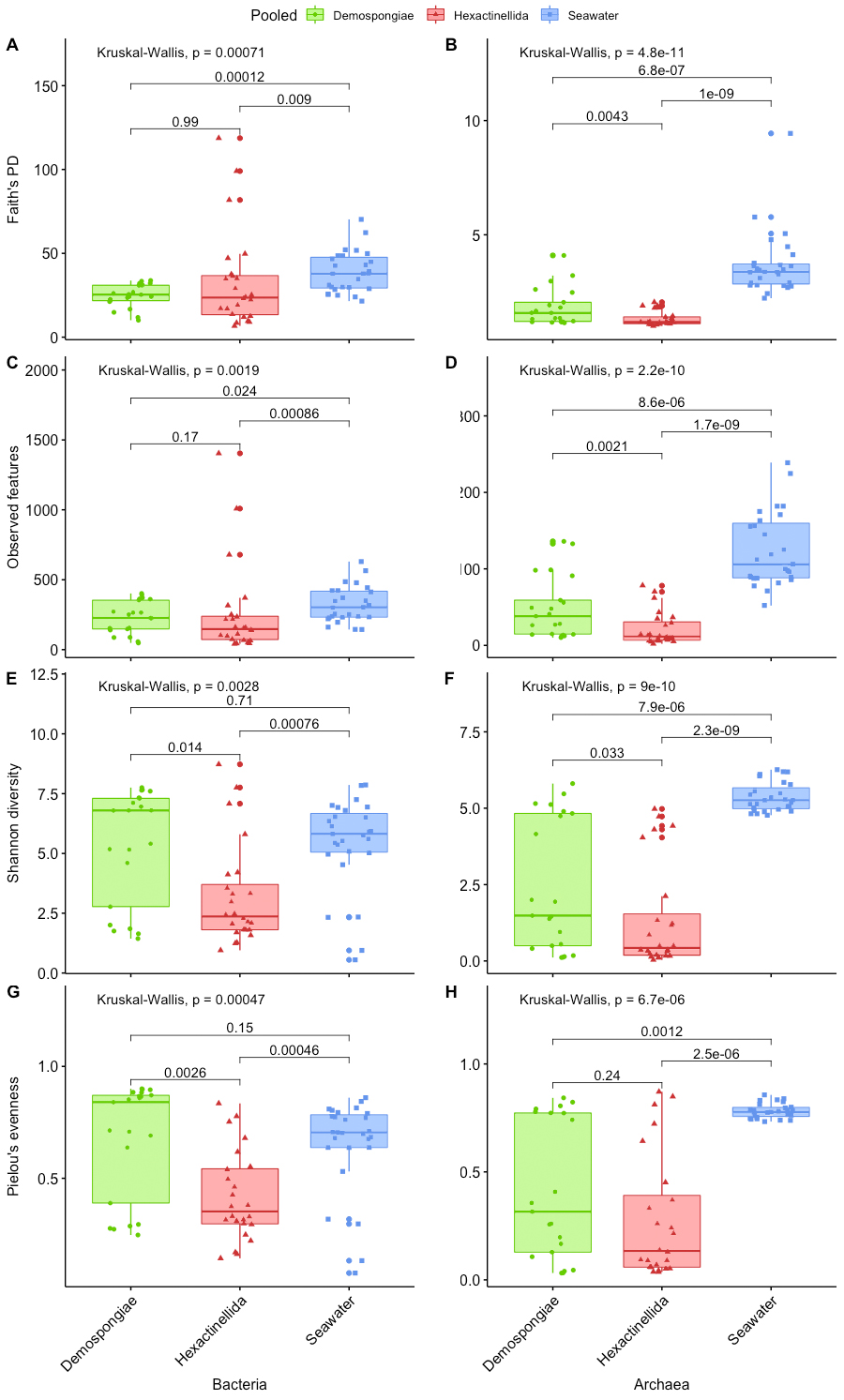

Supplement: FIGURE S2 — Alpha diversity plots of bacterial and archaeal communities. Following indices are shown: (A,B) = Faith’s PD; (C,D) = observed features; (E,F) = Shannon diversity; (G,H) = Pielou’s evenness. [file Image_2.JPEG]

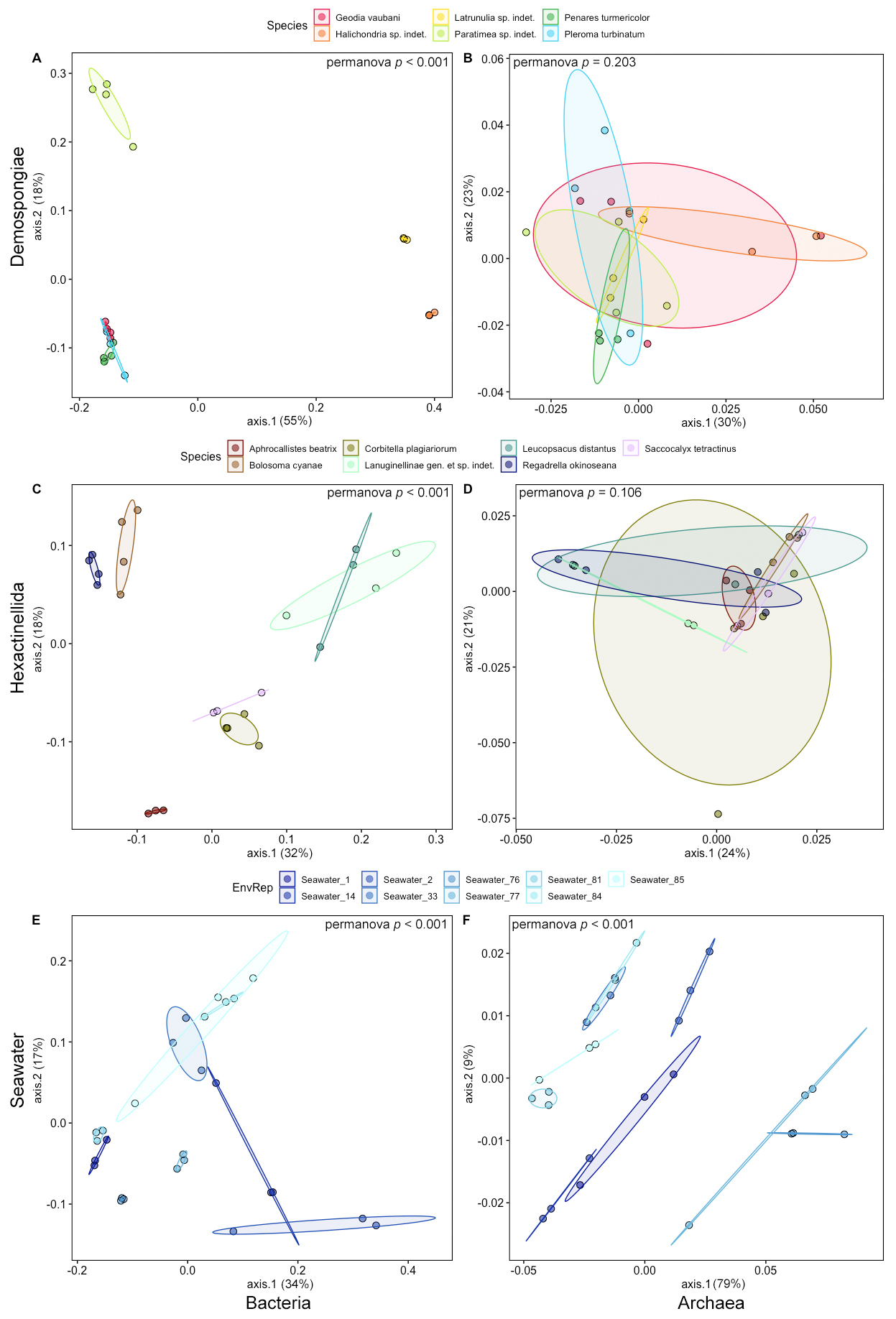

Supplement: FIGURE S3 — Principal component analysis using bacterial and archaeal weighted uniFrac distances for Demospongiae (A,B), Hexactinellida (C,D), and seawater (E,F) samples. [file Image_3.JPEG]

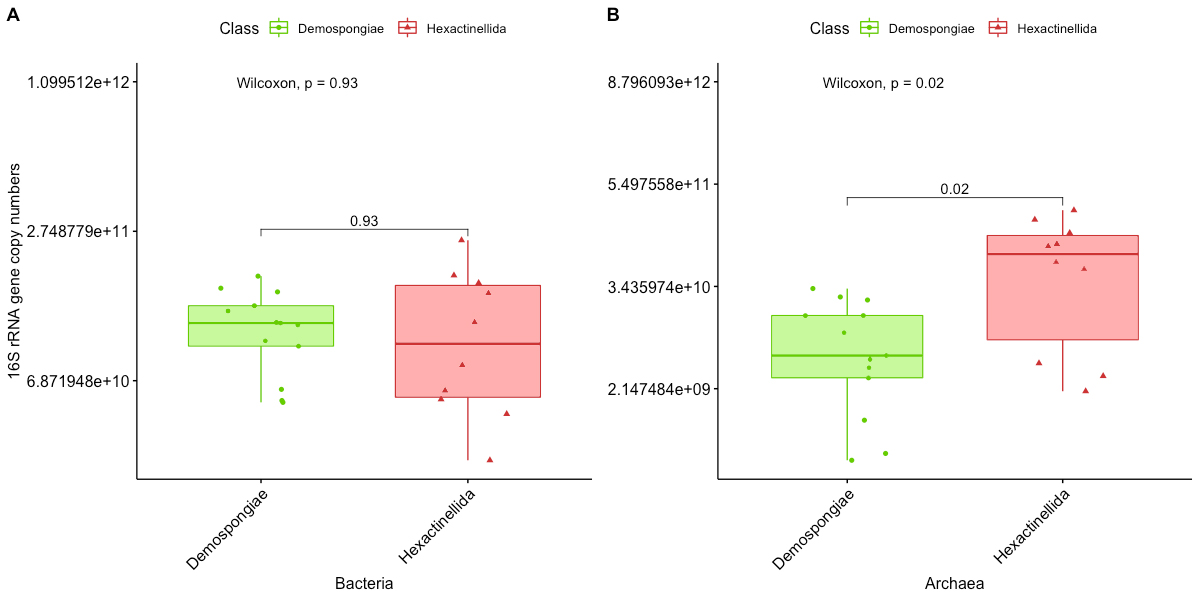

Supplement: FIGURE S4 — 16S rRNA gene copy numbers of (A) bacterial and (B) archaeal quantitative real-time PCR data. [file Image_4.JPEG]
