# Supplementary material for: Compositional and Quantitative Insights Into Bacterial and Archaeal Communities of South Pacific Deep-Sea Sponges (Demospongiae and Hexactinellida)
Source: Front Microbiol. 2020 Apr 24;11:716. doi: 10.3389/fmicb.2020.00716 (PMC7193145; doi:10.3389/fmicb.2020.00716)
Supplement: Supplementary file 9 [file Data_Sheet_1.docx]

**Supplementary Material Section**

**Sampling and amplicon sequencing of bacteria**

Sponges were sampled with the help of the remotely operated vehicle ROV Kiel 6000 (http://jlsrf.org/index.php/lsf/article/view/160). The tissue was subsampled onboard RV SONNE as soon as possible, rinsed to minimize contamination and frozen at -80°C until DNA extraction. Seawater samples (2L) were mainly sampled with Niskin bottles deployed on the ROV and filtered onboard onto PVDF filter membranes (0.22µm pore size and ø47mm) and subsequently stored at -80°C until DNA extraction. DNA of sponges and seawater samples was extracted using the DNeasy Power Soil Kit (Qiagen) on approximately 0.25 g of sponge tissue, 0.25 g of sediment or half a seawater filter. After the quality and quantity of the extracts had been checked (by Nanodrop and gel electrophoresis after a PCR with universal 16S primers), a one-step PCR was performed for amplification of the V3 and V4 variable regions of the 16S rRNA gene (primer pair 341F-806R). A quality check by gel electrophoresis, normalization and pooling was performed on the products before sequencing them on a MiSeq platform (MiSeqFGx, Illumina) with v3 chemistry.

**Archaeal library preparation**

The V46 region (471 bp) of the 16S gene has been amplified for 292 Archaea specimens using the primers Uni519F and Arch1000R (ref). A 4 bp nucleotide barcode has been designed and added to the 5’ end of each primer resulted in unique primer combinations assigned to each PCR plate. The Nextera XT compatible illumina adapter overhang sequence was added to 5’ end of the primers following the Illumina 16S Metagenomic Sequencing Library Preparation guide resulting in following compositions:

5’-TCGTCGGCAGCGTCAGATGTGTATAAGAGACAG‐barcode-Uni519F

5’GTCTCGTGGGCTCGGAGATGTGTATAAGAGACAG‐barcode-Arch1000R.

Two PCRs have been performed to amplify the V46 fragment and incorporate the second part of the Illumina adapters (i7 & i5) and Nextera XT v2 (set A, 96 samples) indexes; the first PCR was performed using the composite primers in a unique barcode combination for each plate and Phusion Green Hot Start II High-Fidelity PCR Master Mix (ThermoFisher) using 2 µl of template DNA and 0.5 µl of each primer (10 pmol) in a final 20 µl reaction volume following the protocol: Initial denaturation at 98°C for 2 minutes and 25 cycles of denaturation at 98°C for 15 seconds, annealing at 57°C for 30 seconds, extension at 72°C for 30 seconds followed by a final extension of 2 minutes at 72°C.

Five µl of the first PCR products have been purified using 2 µl of ExoSAP-IT PCR Product Cleanup Reagent (ThermoFisher) following incubation of 5 minutes at 37°C and 1 minutes at 80°C.

A second PCR using Nextera Indexed primers (dual indexing approach) was performed using 7 µl of the purified first PCR amplicons, 0.5 µl of each index primer (10 pmol) in a final volume of 20 µl, and 15 cycles of a two-step PCR protocol as follow: denaturation at 98°C for 15 seconds, annealing and extension at 72°C for 40 seconds and final extension at 72°C for 2 minutes. The PCR products have been tested for the fusion of the full Illumina adapter to the amplicons comparing the length of the fragments from the 1st (571 bp) and 2nd PCR (640 bp) in a 1.5% Agarose Gel stained with 1% Gel Red. V46 fragment has been successfully amplified and incorporated to Nextera indexes and Illumina adapters in the total number of 236 specimens. Two μl of each amplicon has been pooled prior purification using AMPure XP PCR purification beads (Beckman Coulter) in the ratio of 1:0.7 (beads: amplicons). Qubit 4 Fluorometric Quantification dsDNA High Sensitivity (HS) Assay (Thermofisher) was used to measure the concentration of DNA of the pooled purified library.

The pooled library was diluted to a concentration of 4 nM prior sequencing considering the average amplicon length of 640 bp (insert length of V46 fragment and Nextera adapters). Five μl of the 4 nM library has been denatured into single stranded DNA using 5 μl of freshly prepared NaOH 0.2 M and further diluted into 20 pM using pre-chilled Hyb1 buffer (Illumina). In order to increase the nucleotide diversity in the first sequencing cycles, 20% volume of 20 pM PhiX control was spiked into the library. The spiked Library was further diluted to 14 pM in 600 μl prior sequencing on a MiSeq using V3 chemistry and 301 paired-end read frame. A total of ~19 million reads were produced in which ~ 17 million passed the filter with the quality of 82.3 % >=Q30. The Illumina reads were demultiplexed and renamed addressing the specific barcode of each plate using a custom bash script.
